# Resveratrol therapy modulates inflammatory biomarkers and reverses mechanical allodynia in rats subjected to a temporomandibular joint arthritis model

**DOI:** 10.1007/s10787-026-02183-9

**Published:** 2026-03-31

**Authors:** Iala Thais S. Morais, Vitória Farias de Oliveira, Thaís Collioni de Oliveira, Amanda Stieven, Alanis S. Melo, Wolnei Caumo, Dirson J. Stein, Iraci L.S. Torres

**Affiliations:** 1 Laboratory of Pain Pharmacology and Neuromodulation: Preclinical Investigations, Hospital de Clínicas de Porto Alegre, 90035-903, Ramiro Barcelos, 2350, Porto Alegre, Rio Grande do Sul Brazil; 2 Post-Graduate Program in Biological Sciences: Pharmacology and Therapeutics, Universidade Federal do Rio Grande do Sul, Porto Alegre, RS Brazil; 3 Program in Medicine: Medical Sciences, Faculdade de Medicina, Universidade Federal do Rio Grande do Sul, Porto Alegre, RS Brazil; 4 Translational Nucleus: Pain Pharmacology and Neuromodulation, Hospital de Clínicas de Porto Alegre, Rua Ramiro Barcelos, n. 2350. Bairro Santa Cecília, Porto Alegre, RS 90035-903 Brazil

**Keywords:** Chronic pain, Orofacial pain, Inflammation, Resveratrol, Rats

## Abstract

Toothaches, headaches, and chronic illnesses, including trigeminal neuralgia and temporomandibular joint dysfunction, are common craniofacial pain conditions. Resveratrol, a polyphenolic compound characterized by its antioxidant and anti-inflammatory properties, has shown therapeutic potential across multiple neurological disorders. This study aimed to evaluate the antinociceptive, anti-inflammatory, behavioral, and neurochemical impacts of Resveratrol in rats experiencing chronic inflammatory orofacial pain. A unilateral intra-trigeminal neuralgia injection (50 µL) of Complete Freund’s Adjuvant in adult male Wistar rats was used to induce pain. Resveratrol at 20, 40, or 80 mg/kg was administered to SHAM (saline) and Pain rats for 14 days. Mechanical allodynia (von Frey test) and thermal nociceptive sensitivity (acetone test) were assessed. Cytokine levels (TNF-α, IL-10, and IL-4) were quantified in the spinal cord and brainstem. Animals treated with Resveratrol demonstrated a progressive recovery in mechanical thresholds over time, with RES40 and RES80 groups exhibiting significantly higher thresholds (day 17) compared to the Pain group. Increased grooming time was observed during the acetone test in rats submitted to the pain model (day 3), with no significant effects of treatment (day 17). The pain model significantly increased spinal cord levels of TNF-α and IL-4. All doses of Resveratrol effectively mitigated the elevation in TNF-α levels in the spinal cord, while only RES80 potentiated the increase in IL-4 levels induced by the pain model. Despite limitations related to the pharmacokinetic characteristics of RES, the results indicate that this compound holds translational potential as an adjuvant in the management of painful inflammatory conditions, including TMJ disorders.

## Introduction

Orofacial pain is frequently associated with temporomandibular dysfunction (TMD), a collective term for disorders affecting the temporomandibular joint (TMJ), characterized by abnormal joint sounds, restricted or asymmetric mandibular movements, and pain, primarily localized in the TMJ and masticatory muscles (Conti and De Godoi Gonçalves 2022).

Despite advances in drug evaluation and development for managing orofacial pain, the multiple and complex mechanisms involved in TMD make treatment challenging and frequently unsatisfactory (Klasser and Reyes 2023). In this context, nonsteroidal anti-inflammatory drugs (NSAIDs) are widely used to relieve inflammatory pain associated with TMD. However, managing this disorder requires differentiated therapeutic approaches owing to the diversity of its causes (Wieckiewicz et al. 2015). Thus, it is essential to identify new therapeutic targets and develop more effective interventions for TMD treatment.

Resveratrol (RES), a naturally occurring polyphenolic compound chemically identified as 3,5,4′-trihydroxy toluene, has been extensively investigated for its broad spectrum of biological activities, including cardioprotective, anticancer, and anti-aging effects in multiple organisms (Khorshidi et al. 2020; Meng et al. 2020). These benefits are attributed to the compound’s antioxidant, anti-inflammatory, cell growth-modulating, and anticancer properties (Pinheiro et al. 2018).

Recent studies indicate that RES also exhibits potential for pain modulation, particularly due to its antioxidant and anti-inflammatory characteristics (Dong et al. 2022; Faisal et al. 2024; Wang et al. 2012). It has been demonstrated that RES administration reduces chronic neuropathic pain in mice (Tao et al. 2016), is effective in alleviating inflammatory pain in the TMJ (Ma et al. 2020), and in improving behavioral and pathological alterations in neurological disorders, including trigeminal neuropathic pain in rats (Yang et al. 2016).

Given the complexity and multifactorial nature of chronic orofacial pain, particularly in conditions involving the temporomandibular joint (TMJ), there is a growing need for therapeutic strategies that go beyond conventional analgesics. Current treatments often fail to provide satisfactory long-term relief and are associated with undesirable side effects (Magni et al. 2018). Preclinical studies have demonstrated that the dysregulated expression of pronociceptive and pro-inflammatory mediators, such as TNF-α and IL-1β, contributes significantly to the sensitization of trigeminal pathways and the persistence of TMJ inflammation (Han et al. 2023). In light of this, RES, a natural polyphenol with antioxidant and anti-inflammatory properties, has gained attention as a potential modulator of neuroinflammatory processes in pain models (Wang et al. 2020; Lu et al. 2010). However, its optimal dose and full therapeutic potential in chronic orofacial inflammatory pain models remain to be clarified. Therefore, the present study was designed to investigate the dose-dependent effects of RES on behavioral, inflammatory, and neurochemical parameters in rats submitted to a chronic inflammatory orofacial pain model.

## Materials and methods

### Animals

Forty-eight adult male Wistar rats (3–4 months old), weighing between 300 and 500 g were obtained from the Laboratory Animal Reproduction and Experimentation Center (CREAL) at the Universidade Federal do Rio Grande do Sul (UFRGS). Animals were group-housed (three per cage) in polypropylene cages (49 × 34 × 16 cm) with a bedding layer of wood shavings. All rats were maintained in a controlled environment (22 ± 2° C, 40–60% air humidity) under a standard 12-hour light-dark cycle (lights on at 7 a.m.), with rodent chow (Nuvilab^®^, Quimtia Brasil, Colombo - PR/Brazil) and water provided *ad libitum.* All experiments and procedures were approved by the Institutional Animal Care and Use Ethics Committee (protocol GPPG-HCPA #19.0763) and followed the guidelines of the Guide for the Care and Use of Laboratory Animals, 8th ed. (2011). Animal maintenance complied with Law #11,794, which regulates the scientific use of animals in Brazil. The experimental protocol adhered to the ethical and methodological standards of the ARRIVE guidelines (Kilkenny et al. 2010).

### Experimental design

**Fig. 1:**
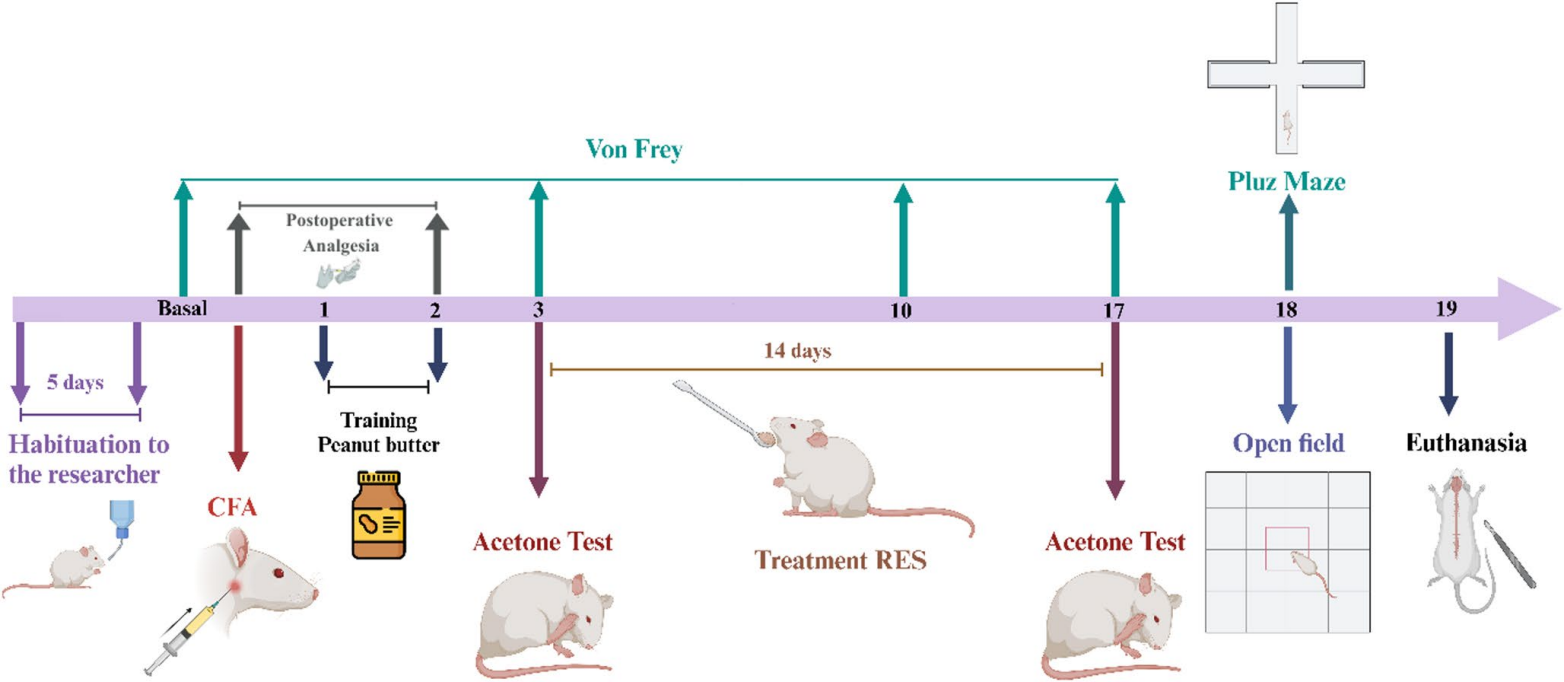
Experimental Timeline. Rats were habituated to the experimental facility for 14 days and to the investigators for 5 days prior to the start of the procedures. Complete Freund’s Adjuvant (CFA) or saline solution was injected on Day 0. Every 12 h following CFA administration, rats received tramadol hydrochloride (20 mg/kg, intraperitoneally) twice daily from Day 0 to Day 2 to manage postoperative pain. From Days 1 to 3, animals were habituated to peanut butter administration. Resveratrol (RES) was administered daily from Day 3 to Day 17. Facial mechanical sensitivity was assessed using electronic von Frey testing on the day prior to CFA injection (baseline) and on Days 3, 10, and 17. The acetone test for cold allodynia was performed on Days 3 and 17. The open field and elevated plus maze tests were conducted on Day 18. On Day 19, all animals were euthanized, and biological samples were collected for biochemical analyses

Upon arrival, the rats underwent a 14-day environmental habituation period to the animal facility (Animal Experimentation Unit, Hospital de Clínicas de Porto Alegre - HCPA), and habituation to investigators handling for 5 days. Prior to the induction of the inflammatory pain model (Day 0), the animals were first assigned to three groups, balanced according to body weight and baseline von Frey data, as follows: (1) Control, (2) SHAM (S), and (3) Pain (P). After 14 days of the pain induction model, the Pain group was subdivided into (1) Pain (P); (2) Pain + RES 20 mg/kg (RES-20), (3) Pain + RES 40 mg/kg (RES-40), and (4) Pain + RES 80 mg/kg (RES-80). The Control and SHAM groups exhibited no differences regarding nociceptive levels, thus only the Control group was considered in statistical analysis, resulting in a total of 5 groups. Initially, on the first experimental day (D0), the animals received saline (SHAM group) or Complete Freund’s Adjuvant (CFA) (Pain groups) into the right TMJ. The SHAM group did not undergo any treatment and served as a control for the pain model. A dose-response curve for RES was established to determine the most effective dose for pain treatment. Treatment began on the third day post-CFA (D3) and lasted for 14 days, with different doses of RES administered orally (PO). Behavioral tests were conducted at different time points throughout the study, allowing for a temporal assessment of inflammatory pain and treatment effects. The nociceptive responses, mechanical allodynia (von Frey test) and thermal nociceptive response (Acetone test) were used as the primary outcome, and other behaviors (Open Field and Elevated Plus Maze tests) and neurochemical parameters were used as the secondary outcomes. The von Frey test was conducted at baseline, D3, D10, and D17 after model induction. Rats were tested on the Open Field (D18), on the Elevated Plus Maze (D18). The number of animals used for this experiment was calculated to produce reliable scientific data. The animals were randomly tested to maintain experimenter blinding. Finally, 24 h after the end of the behavioral tests (D19), the animals were euthanized by decapitation using a rodent guillotine, and the trigeminal nerve and ganglion and TMJ were harvested for biochemical assays (Fig. 1).

### Orofacial pain model induced by complete Freund’s Adjuvant (CFA)

CFA is a widely used compound for studying inflammatory pain in rodents, as it induces intense and persistent pain while promoting a biphasic inflammatory reaction: an initial phase (acute inflammation) and a late phase (chronic inflammation, considered from the fourteenth day post-CFA injection). First, the rats were anesthetized using vaporized isoflurane (5% for induction, 2% for maintenance). Chronic inflammatory orofacial pain was induced by intra-articular administration of 50 µL CFA (1.0 mg/mL *Mycobacterium tuberculosis*, F5881, Sigma-Aldrich, St. Milan, Italy) into the right TMJ. Previous studies have demonstrated that this dose of CFA, when injected into the TMJ, leads to persistent behavioral hyperalgesia. Animals in the Sham group received 50 µL of a 0.9% sterile saline solution in the right TMJ. The TMJ region was identified by palpation, and CFA or saline injection was performed manually by advancing a 30-gauge needle through the skin, just below the posterior border of the zygomatic arch, until it touched the mandibular condyle. Immediately after the intra-TMJ injection, and every 12 h thereafter, for three days (from Day 0 to Day 2), all rats received tramadol hydrochloride (20 mg/kg, i.p.) twice daily for pain relief (Kroeff et al. 2025).

### Pharmacological treatments

For treatments, animals received either vehicle (peanut butter − 1 g/kg) (Warren et al. 2020) or RES combined with the vehicle at 20, 40, and 80 mg/kg doses. The repeated treatment protocol consisted of oral (PO) administration for 14 days, beginning on the third day after pain model induction. Voluntary oral administration is preferred for its ability to mitigate stress responses. The cellular immune response and antibody production triggered inflammation within 3–5 days (Sadighparvar et al., 2023), thus supporting the decision to start treatment 3 days after induction.

### Behavioral tests

Following the end of RES treatment, the behavioral tests were conducted over two days to mitigate potential bias resulting from prior exposure to other tests. On days when different behavioral tests were conducted, a minimum interval of 6 h was observed. Nociceptive tests, being more susceptible to external interventions, were conducted on day 17, following the completion of repeated doses of RES at different times throughout the day: the von Frey test in the morning and the acetone test in the afternoon. On day 18, the animals were subjected to behavioral evaluations using the Elevated Plus Maze test in the morning and the Open Field test in the afternoon.

#### Mechanical Allodynia (Electronic facial von Frey test)

The mechanical nociceptive threshold of the TMJ region was assessed using an electronic von Frey (VF) algometer (model EFF301, Insight, São Paulo, Brazil), measuring the latency for head withdrawal in response to applied pressure (intensity in grams - g). The test is based on the maximum pressure required for the animal to show sensitivity to skin contact. All test sessions were conducted by the same trained investigator, who was blinded to the treatment groups. To minimize stress and novelty-induced analgesia, 24 h before testing, the animals were acclimated to the testing environment and the restraint process using a soft cloth for 10 min. The von Frey test was conducted at baseline and repeated on days 3 (D3), 10 (D10), and 17 (D17) after pain model induction, always in the morning. On the test day, the animals were transferred to the testing room 1 h in advance for acclimatization. During the test, the animals were manually restrained using a soft cloth, and the sensor of the algometer was positioned over the TMJ region. The pressure was gradually increased until a facial withdrawal response or aversive vocalization was observed. For each rat, three measurements were taken on the left and right sides of the face, and the average was considered as the animal’s mechanical nociceptive threshold. Testing was always conducted in the morning, before the daily treatment dose. The average of the three measurements was considered the animal’s mechanical nociceptive threshold.

#### Thermal nociceptive threshold (acetone test)

Cold allodynia was assessed using the acetone test as established by Ma et al., adapted for rats. The test consists of applying 50 µL of acetone to the extraoral surface in the vibrissae area, ipsilateral to the CFA injection site, using a 30 IU insulin syringe with a blunt needle, ensuring that the needle does not touch the animal’s skin. After acetone application, each animal was individually placed in an acrylic box, and behavior was videorecorded for one minute and further evaluated for the frequency of facial grooming (Ma et al. 2012). This test was conducted on the first (D3) and last day (D17) of treatment, both in the afternoon.

#### Open field test

The open field (OF) test assessed animal locomotion and exploration. The assessment was conducted in a varnished wooden box of 50 × 60 × 40 cm with a glass front wall and linoleum floors separated into 12 rectangles of 15.0 × 13.3 cm with dark lines. The animals were gently placed in the back left corner of the box and permitted to explore for 5 min (Gould 2009). Blind researchers assessed videorecorded behaviors. The animal’s latency to leave the first square—from placing all four paws in the next quadrant—was considered an indicator of anxious-like behavior. The number of crossings was used to assess locomotion (Britton and Britton 1981). Exploratory activity was measured by rearings, or elevating both front paws. Grooming was used as a measure of biological self-cleaning and anxiety-like behavior (Nin et al. 2012). Evaluations were conducted 24 h following the end of treatment (D18) in the afternoon.

#### Elevated plus maze test

The elevated plus maze (EPM) was used to assess the animal’s anxious-like behavior. The apparatus consists of two open arms and two closed arms (50 × 40 × 10 cm) extending from a common central platform (10 × 10 cm). At the beginning of the test, the animal was placed in the central area of the apparatus, facing one of the open arms, and its behavior was videorecorded for 5 min. Behaviors were evaluated by blind investigators, as follows: (1) number of entries into the open arms (EOA); (2) number of entries into the closed arms (ECA); (3) total number of entries (TE); (4) time spent in the open arms (TOA); (5) time spent in the closed arms (TCA), which was considered to reflect the fear of entering the open areas, associated with rat anxiety (Benetti et al. 2007) ; (6) number of protected head dips (PHD); (7) number of unprotected head dips (UHD), indicative of exploratory activity; (8) grooming time (in seconds); (9) number of rearings; (10) anxiety index, calculated using the following formula: 1 - ((TOA/300) + (EOA/TE) / 2 (Dornellas et al. 2018). This test was conducted 24 h after the treatment ended (D18) in the morning.

### Tissue collection

The animals were euthanized by decapitation one day after the end of the behavioral tests (D19; Fig. 1). The spinal cord and brainstem were dissected, collected, and stored at − 80 °C for later analysis.

### Neurochemical analyses

Initially, tissue samples were processed with a tissue homogenizer (Ultra Stirrer, Ultra 380) using protease inhibitor cocktail (Roche Diagnostics, Cat. #11836153001) diluted 1:100 in Phosphate Buffered Saline (PBS). The spinal cord and brainstem TNF-α, IL-10, and IL-4 levels were evaluated using the Enzyme-Linked Immunosorbent Assay (ELISA) technique with DuoSet ELISA kits, employing specific monoclonal antibodies (R&D Systems, Minneapolis, USA; Cat. #DY510 / Lot #P362517 for TNF-α, Cat. #DY522 / Lot #P354575 for IL-10, and Cat. #DY504 / Lot #P354859 for IL-4). The assays were carried out according to the manufacturer’s instructions. Optical density was measured using an ELISA reader at a wavelength of 450 nm (SpectraMax M3, Molecular Devices factory calibrated). Total protein was determined using the Bradford method, with bovine serum albumin as the standard (Bradford 1976). The data were expressed in pg/mg of protein.

### Statistical analyses

All results were subjected to a homogeneity analysis using the Levene’s test and to a normality of distribution analysis using the Shapiro-Wilk test and histograms to determine the appropriate statistical method to be employed. ANOVA and Kruskal-Wallis tests are employed as methods for multiple comparisons among independent groups. *Post hoc* testing is utilized to adjust the significance level, which improves the accuracy of the results. Data that followed a normal distribution were analyzed using parametric tests (one-way or repeated measures ANOVA). In this example, *post hoc* comparisons were conducted employing the Bonferroni method when an interaction between Time and Group was observed, as was the case with the von Frey data. Duncan’s *post hoc* approach was employed when only group effects were observed, as was the case with the behavioral assessments. To compare the strength and direction of an association between the study variables, the Spearman’s rank correlation was employed. Statistical analyses were performed using SPSS (Statistical Package for the Social Sciences), version 18.0. Data were expressed as mean ± standard deviation (SD) or median ± interquartile range (IQR), considering *P* < 0.05 for statistically significant differences.

## Results

### Mechanical nociception

**Fig. 2:**
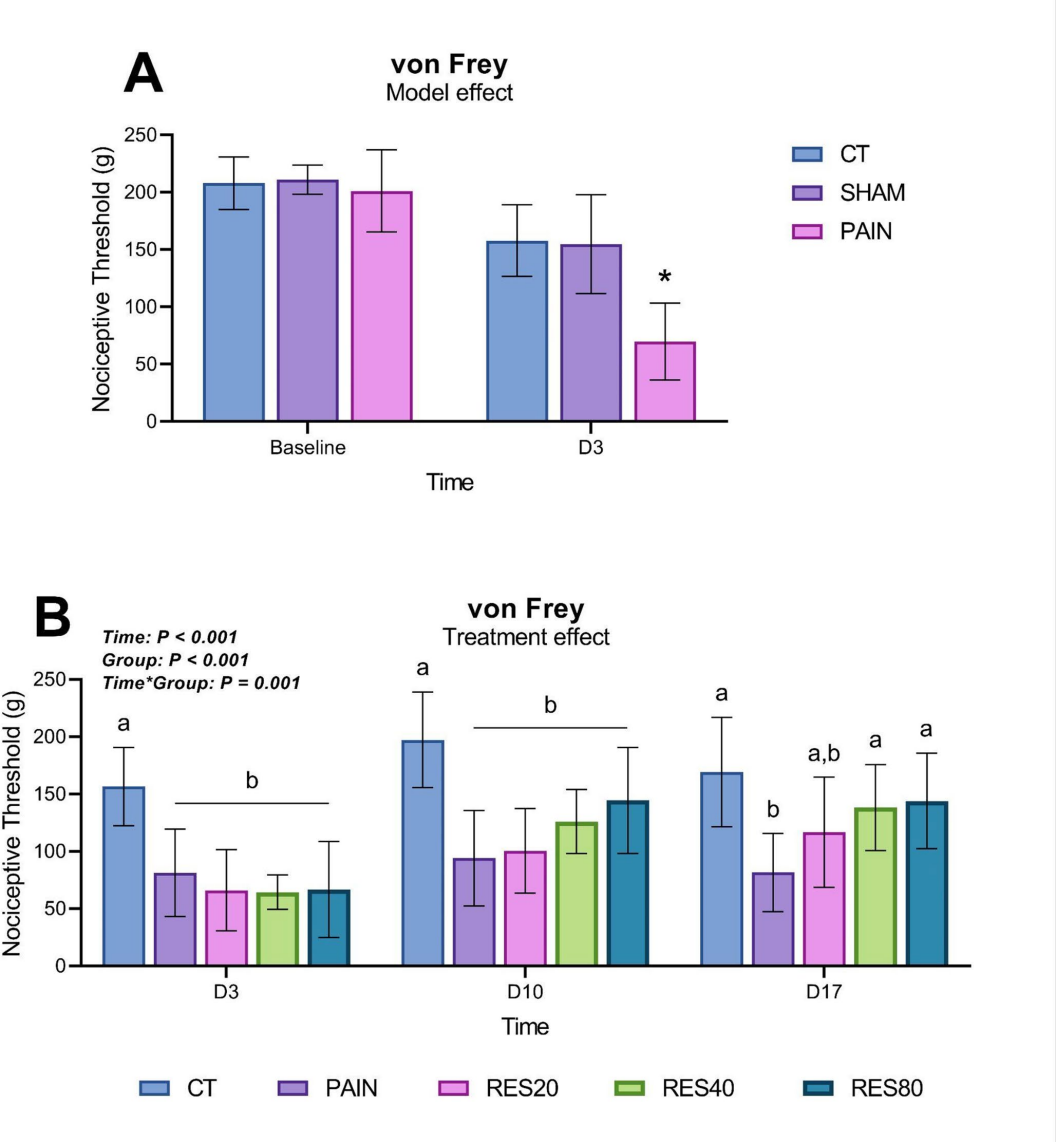
Mechanical nociceptive threshold assessed through the electronic von Frey test. Data are presented as mean ± SD. Panel A: Pain model effect at baseline and at D3 [*n* = 6 (CT), 4 (Sham), and 39 (Pain)]. There were no significant differences between groups at baseline nociceptive threshold (Bonferroni, *P* = 1.000). * indicates a statistically significant difference from the CT and Sham groups at D3 (Bonferroni, *P* < 0.001). Panel B: Mechanical nociceptive threshold at D3 (pre-treatment), D10 (during treatment), and D17 (after treatment). This figure shows the maintenance of the pain model and the effect of repeated RES treatment in different doses [after treatment - *n* = 10 (CT), *n* = 9 (Pain), *n* = 9 (RES20), *n* = 10 (RES40) and *n* = 10 (RES80)]. Same letters indicate no significant differences between groups: a = a, b = b (Bonferroni, *P* > 0.05); Different letters indicate statistically significant differences between groups: a ≠ b (Bonferroni, *P* < 0.05)

To assess the effects of saline or CFA injection (Sham and pain model, respectively) on mechanical allodynia, groups were compared at baseline and on D3. The repeated measures ANOVA shows an effect of Time (*F*_(1)_ = 53,765; *P* < 0.001), an effect of Group (*F*_(2)_ = 16,262; *P* < 0.001), and a Time*Group interaction (*F*_(2)_ = 10,441; *P* = 0.001; Fig. 2A). Multiple *post hoc* comparisons showed that the CT, Sham, and Pain groups were similar at baseline (Bonferroni, *P* = 1.000), yet the Pain group was significantly different from the CT and Sham groups at D3 (Bonferroni, *P* < 0.001), thereby confirming the diminished mechanical nociceptive threshold of the Pain group and the establishment of the pain model.

To assess the effects of the pain model over time and of treatment with different doses of RES on mechanical allodynia, all groups were compared at D3, D10, and D17 using repeated measures ANOVA. Given that the CT and Sham groups exhibited no differences regarding their nociceptive thresholds, these animals were combined into a single group (CT) for statistical analysis (Fig. 2A). The test revealed an effect of Time (*F*_(2)_ = 41,142; *P* < 0.001), an effect of Group (*F*_(4)_ = 12,213; *P* < 0.001) and a Time*Group interaction (*F*_(8)_ = 4,427; *P* = 0.001; Fig. 2B). Multiple *post hoc* comparisons showed that all groups injected with CFA exhibited reduced mechanical nociceptive thresholds compared to the CT group on D3 and D10 (Bonferroni *P* < 0.05). At D17, the Pain group was significantly different from the CT (Bonferroni *P* < 0.001), suggesting a persistent state of hypersensitivity in nontreated animals. The RES20 group exhibited a partial reversion, being similar to both the CT (Bonferroni *P* = 0.095) and Pain (Bonferroni *P* = 0.751) groups, whereas the RES40 and RES80 groups were different from the Pain group (Bonferroni *P* = 0.042 and *P* = 0.018, respectively) and similar to the CT group (Bonferroni *P* = 1.000 for both), showing a complete reversion in mechanical allodynia induced by the pain model.

### Thermal nociception

**Fig. 3:**
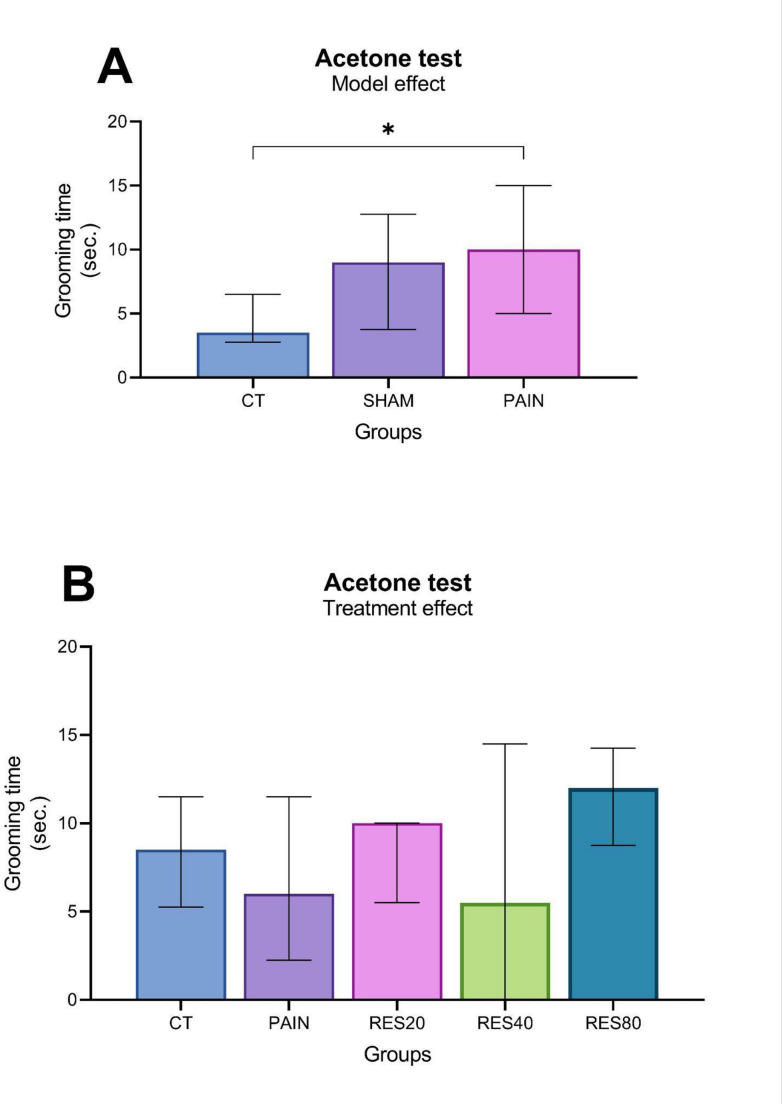
Grooming time assessed through the Acetone test. Data were expressed in seconds, analyzed by Kruskal-Wallis, and presented as median ± interquartile range (IQR). *n* = 9 to 10/group). Panel A: Pain model effect at D3 [*n* = 6 (CT), 4 (Sham), and 39 (Pain)]. * There was a significant statistical difference from the CT group (Dunn, adjusted *P* = 0.034). Panel B: Treatment effect at D17 [after treatment - *n* = 10 (CT), *n* = 9 (Pain), *n* = 9 (RES20), *n* = 10 (RES40) and *n* = 10 (RES80)]. There was no significant difference between groups (Kruskal-Wallis, *P* = 0.202)

To assess the effects of saline or CFA injection (Sham and pain model, respectively) on thermal hyperalgesia (Acetone test), the CT, Sham and Pain groups were compared at D3. The Kruskal-Wallis test indicated a statistically significant difference between groups (*H*_(2)_ = 6,448; *P* = 0.040). A *post-hoc* pairwise comparison using Dunn’s test shows that the Pain group displayed longer grooming time than the CT group (adjusted *P* = 0.034; Fig. 3A), suggesting an increased nociceptive sensitivity. The Sham group was similar to both the CT and the Pain groups (Dunn *P* > 0.05 for both).

To assess the effects of the pain model over time and of treatment with different doses of RES on thermal hyperalgesia, all groups were compared at D17 using the Kruskal-Wallis test. Given that the CT and Sham groups exhibited no differences regarding grooming time, these animals were combined into a single group (CT) for statistical analysis. No significant differences were observed between groups after the end of treatment (*H*_(4)_ = 5,965; *P* = 0.202). These results demonstrated a reversal of the initial thermal hypersensitivity observed at D3.

### Locomotor and exploratory activities

To assess the effects of the pain model and of treatment with different doses of RES on locomotor and exploratory activities in the OF test conducted on D18, all groups were compared using one-way ANOVA/Duncan (Table 1). No significant differences were detected for the latency to exit the first quadrant (*F*_(4)_ = 2,226; *P* = 0.082), latency to access the center (*F*_(4)_ = 1,555; *P* < 0.203), frequency of crossings in the center (*F*_(4)_ = 1,265; *P* = 0.298), and periphery (*F*_(4)_ = 0,375; *P* = 0.825), total crossings (*F*_(4)_ = 0,479; *P* = 0.751), time in the center (*F*_(4)_ = 1,454; *P* = 0.233), time in the periphery (*F*_(4)_ = 1,568 ; *P* = 0.200), frequency of rearings (*F*_(4)_ = 0,510; *P* = 0.728), and grooming duration (*F*_(4)_ = 1,555; *P* < 0.203).

**Table 1:** Open Field test

Variable	CT (*n* = 10)	Pain (*n* = 9)	RES20 (*n* = 9)	RES40 (*n* = 10)	RES80 (*n* = 10)	*P*-value
Latency first quadrant (sec.)	2.90 (± 2.18)	4.33 (± 3.39)	2.78 (± 2.04)	2.10 (± 0.87)	4.70 (± 2.45)	0.082
Latency to center (sec.)	19.50 (± 21.64)	22.00 (± 27.67)	14.00 (± 13.10)	40.20 (± 28.13)	40.00 (± 48.01)	0.203
Center crossings (n)	13.80 (± 5.99)	15.88 (± 3.72)	14.77 (± 5.42)	14.70 (± 6.00)	10.90 (± 6.48)	0.298
Periphery crossings (n)	81.90 (± 11.67)	94.66 (± 28.18)	90.00 (± 23.84)	89.80 (± 19.50)	87.30 (± 30.18)	0.825
Total crossings (n)	95.70 (± 13.68)	110.55 (± 30.90)	103.66 (± 26.84)	105.50 (± 22.42)	98.20 (± 33.85)	0.751
Time in center (sec.)	18.00 (± 9.03)	20.88 (± 8.22)	19.66 (± 13.72)	20.80 (± 9.71)	11.30 (± 10.57)	0.233
Time in the periphery (sec.)	282.00 (± 9.03)	279.11 (± 8.22)	279.66 (± 12.87)	279.20 (± 9.71)	288.70 (± 10.57)	0.200
Rearing (n)	41.60 (± 10.40)	42.88 (± 8.69)	46.00 (± 12.93)	45.00 (± 6.00)	40.50 (± 10.63)	0.728
Grooming time (sec.)	1.80 (± 1.13)	1.11 (± 1.05)	1.11 (± 1.16)	1.00 (± 1.33)	1.40 (± 1.07)	0.551

Data are presented as mean ± standard deviation (± SD). n – absolute frequency; p – statistical significance; sec - seconds. CT - control; RES – Resveratrol. There were no significant differences between groups in any OF variables evaluated (one-way ANOVA, *P* > 0.05)

### Anxiety-like behavior

To assess the effects of the pain model and of treatment with different doses of RES on anxiety-like behavior in the PM test conducted on D18, all groups were compared using one-way ANOVA/Duncan (Table 2). No significant differences were detected regarding the latency to enter open (*F*_(4)_ = 1,470; *P* = 0.228) and closed arms (*F*_(4)_ = 0,837; *P* = 0.509), time in open (*F*_(4)_ = 2,77; *P* = 0.100) and closed arms (*F*_(4)_ = 2,015; *P* = 0.109), total number of entries (*F*_(4)_ = 0,713; *P* = 0.588), time in the center (*F*_(4)_ = 1,920; *P* = 0.124), frequency of entries into open (*F*_(4)_ = 1,418; *P* = 0.244) and closed arms (*F*_(4)_ = 0,593; *P* = 0.670), UHD (*F*_(4)_ = 0,236; *P* = 0.916), PHD (*F*_(4)_ = 0,808; *P* = 0.527), frequency of rearings (*F*_(4)_ = 1,732; *P* = 0.160), grooming duration (*F*_(4)_ = 0,365; *P* = 0.832), and anxiety index (*F*_(4)_ = 2,149; *P* = 0.091).

**Table 2:** Elevated Plus Maze test

Variable	CT (*n* = 10)	Pain (*n* = 9)	RES20 (*n* = 9)	RES40 (*n* = 10)	RES80 (*n* = 10)	*P*-value
Latency to open arm (sec.)	28.00 (± 48.20)	15.66 (± 25.44)	45.55 (± 85.66)	15.40 (± 22.17)	43.80 (± 71.88)	0.636
Latency to closed arm (sec.)	33.70 (± 43.50)	16.00 (± 11.50)	13.77 (± 16.31)	21.70 (± 17.15)	14.00 (± 14.13)	0.324
Time in open arms (sec.)	58.50 (± 46.95)	50.00 (± 30.73)	31.44 (± 33.59)	78.50 (± 60.61)	46.20 (± 41.36)	0.231
Time in closed arms (sec.)	140.20 (± 46.98)	179.55 (± 44.81)	189.44 (± 39.18)	146.90 (± 59.69)	185.20 (± 56.67)	0.109
Time in center (sec.)	101.30 (± 55.42)	70.44 (± 43.91)	79.11 (± 27.21)	74.60 (± 20.50)	70.30 (± 32.35)	0.352
Entries in open arms (n)	3.10 (± 1.66)	3.66 (± 2.00)	1.77 (± 1.56)	3.20 (± 1.87)	2.60 (± 1.95)	0.244
Entries in closed arms (n)	9.70 (± 2.83)	9.88 (± 3.10)	9.66 (± 2.91)	8.60 (± 5.59)	8.20 (± 3.76)	0.670
Total number of entries (n)	12.80 (± 3.61)	13.55 (± 5.50)	11.44 (± 3.57)	11.80 (± 2.7)	10.80 (± 5.16)	0.588
UHD (n)	8.00 (± 4.42)	6.66 (± 3.04)	7.00 (± 4.21)	8.10 (± 5.15)	6.80 (± 4.66)	0.916
PHD (n)	6.80 (± 2.48)	5.11 (± 3.21)	6.11 (± 1.53)	5.20 (± 2.09)	5.90 (± 2.42)	0.527
Rearing (n)	15.00 (± 4.05)	15.88 (± 4.98)	20.00 (± 4.55)	14.80 (± 5.88)	15.50 (± 5.03)	0.160
Grooming time (sec.)	0.80 (± 0.63)	0.66 (± 0.70)	1.11 (± 1.05)	0.90 (± 0.87)	0.90 (± 0.73)	0.832
Anxiety index	0.78 (± 0.13)	0.79 (± 0.10)	0.89 (± 0.08)	0.74 (± 0.13)	0.82 (± 0.13)	0.091

Data are presented as mean ± standard deviation (± SD). n – absolute frequency; p – statistical significance; sec - seconds. CT - control; Res – Resveratrol. UHD - unprotected head dips; PHD - protected head dips. There were no significant differences between groups in any EPM variables evaluated (one-way ANOVA, *P* > 0.05)

### Biochemistry data

**Fig. 4:**
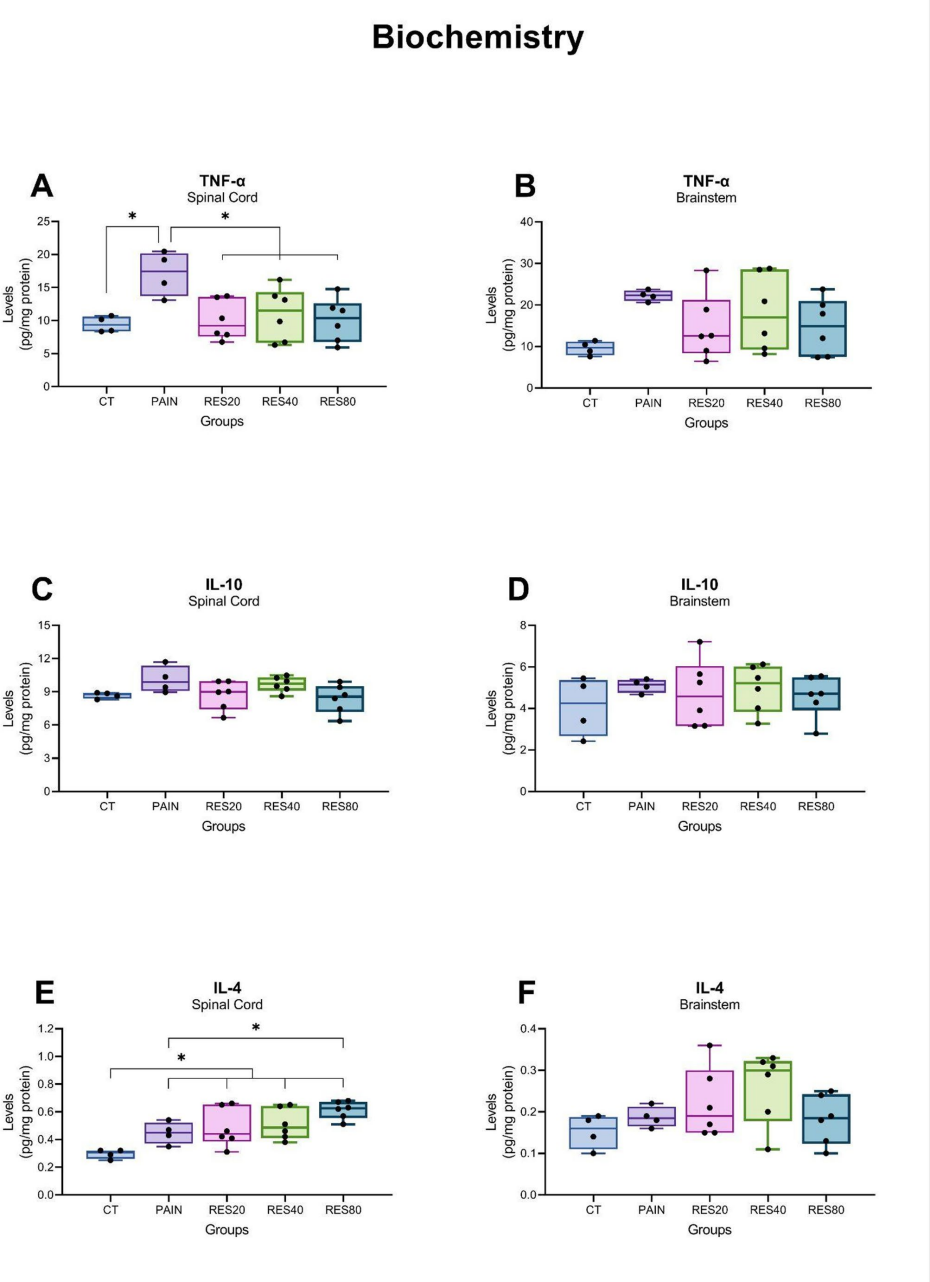
Neurochemical levels of cytokines. Data were expressed in pg/mg protein, analyzed by one-way ANOVA, and presented as median ± interquartile range (IQR) [*n* = 4 (CT), *n* = 4 (Pain), *n* = 6 (RES20), *n* = 6 (RES40) and *n* = 6 (RES80)]. In Panels (**A**) and (**F**), * represents a statistically significant difference between groups (Duncan, *P* < 0.05). In the remaining panels (**B**, **C**, **D**, and **F**), there were no significant differences between groups (one-way ANOVA, *P* > 0.05). **(A)** levels of TNF-α in the spinal cord; **(B)** levels of TNF-α in the brainstem; **(C)** levels of IL-10 in the spinal cord; **(D)** levels of IL-10 in the brainstem; **(E)** levels of IL-4 in the spinal cord; **(F)** levels of IL-4 in the brainstem

To assess the effects of the pain model and of treatment with different doses of RES on central inflammatory markers, the levels of TNF-α, IL-10, and IL-4 were evaluated in the brainstem and spinal cord. The sample from each group that had detectable marker levels within the limits of the ELISA kit curves were used. The one-way ANOVA revealed a statistically significant difference between groups for TNF-α (*F*_(4)_ = 4,037; *P* = 0.014; Fig. 4A) and IL-4 (*F*_(4)_ = 6,448; *P* = 0.002; Fig. 4E) in the spinal cord. *Post hoc* comparisons indicated a significant increase in the spinal cord levels of TNF-α and IL-4 induced by the inflammatory orofacial pain when compared to the CT group (Duncan, *P* < 0.05). All RES doses reverted the increase in TNF-α levels induced by the pain model (Duncan, *P* < 0.05, for all). Treatment with 80 mg/kg/day RES enhanced the effect of pain on the levels of IL-4 (Duncan, *P* < 0.05). No significant differences were observed for the levels of IL-10 in the spinal cord (Fig. 4C) and for the levels of TNF-α, IL-10, and IL-4 in the Brainstem (*P* > 0.05 for all analyses; Fig. 4B and D, and 4F).

### Correlations

The strength and direction of associations between the study variables were assessed using the Spearman’s rank correlation. It shows that several pairs of variables are significantly associated, from which, some are relevant to the study aims. Both positive and negative statistically significant correlations are depicted in Table 3.

A significantly negative association was observed between the mechanical nociceptive threshold at D3 and grooming time in the Acetone test at D3 (R^2^ = − 0.351, *P* = 0.013). Furthermore, the grooming time in the Acetone test at D3 was positively associated with the levels of IL-4 in the spinal cord (R^2^ = 0.613, *P* = 0.001).

Additionally, the biochemical variables from both central structures were associated with each other. The levels of TNF-α in the spinal cord were positively associated with the levels of TNF-α in the brainstem (R^2^ = 0.889, *P* < 0.001), with the levels of IL-10 in the spinal cord (R^2^ = 0.462, *P* = 0.020), with the levels of IL-10 in the brainstem (R^2^ = 0.596, *P* = 0.002), and with the levels of IL-4 in the spinal cord (R^2^ = 0.413, *P* = 0.040). Moreover, the levels of TNF-α in the brainstem were positively associated with the levels of IL-10 in the spinal cord (R^2^ = 0.554, *P* = 0.004), with the levels of IL-10 in the brainstem (R^2^ = 0.542, *P* = 0.001), and with the levels of IL-4 in the spinal cord (R^2^ = 0.512, *P* = 0.009).

Positive associations were also detected between the levels of IL-10 in the spinal cord and the levels of IL-10 in the brainstem (R^2^ = 0.545, *P* = 0.005), between the levels of IL-10 in the brainstem and the levels of IL-4 in the spinal cord (R^2^ = 0.503, *P* = 0.010), and between the levels of IL-10 and IL-4 in the brainstem (R^2^ = 0.461, *P* = 0.020).

**Table 3:** Most relevant correlations between the study variables using the Spearman’s rank coefficient

Correlated variables			Spearman	
			*R* ^2^	*P*
VF D3	X	Grooming D3	− 0.351	0.013
Grooming D3	X	IL-4 SC	0.613	0.001
TNF-α SC	X	TNF-α BS	0.889	< 0.001
TNF-α SC	X	IL-10 SC	0.462	0.020
TNF-α SC	X	IL-10 BS	0.596	0.002
TNF-α SC	X	IL-4 SC	0.413	0.040
TNF-α BS	X	IL-10 SC	0.554	0.004
TNF-α BS	X	IL-10 BS	0.542	0.001
TNF-α BS	X	IL-4 SC	0.512	0.009
IL-10 SC	X	IL-10 BS	0.545	0.005
IL-10 BS	X	IL-4 SC	0.503	0.010
IL-10 BS	X	IL-4 BS	0.461	0.020

Data from the Spearman correlation rank showing the significant association between several pairs of variables. BS = Brainstem; SC = Spinal Cord; VF = von Frey (*n* = 9 to 10/group); Grooming (*n* = 9 to 10/group); TNF-α, IL-10, and IL-4 (*n* = 4 to 6/group)

## Discussion

This study shows relevant effects induced by the TMJ arthritis model in rats. Starting on the third day following the intra-TMJ CFA injection, the animals exhibited a significant reduction in facial mechanical nociceptive thresholds and increased grooming time (indicative of mechanical and cold allodynia, respectively), thereby demonstrating the effectiveness of the orofacial pain model. Nonetheless, mechanical allodynia persisted until at least day 17, which was completely ameliorated by RES at dosages of 40 and 80 mg/kg/day and partially alleviated at 20 mg/kg/day. Furthermore, the arthritic rats exhibited a significant increase in central TNF-α levels, which was fully reversed by RES across all tested dosages. Conversely, central levels of IL-4 were increased by the pain model, with a boost observed only in animals receiving the highest dose (RES80).

Orofacial mechanical and cold allodynia may coexist; however, they originate from distinct processes and have specific features concerning inflammation. The administration of CFA into the joint induces both acute and persistent inflammation, with the extent of deterioration determined by its duration of exposure (Xiang et al. 2021). In addition, the complex interplay between peripheral and central nervous system (CNS) mechanisms contributes to both acute and chronic pain manifestations (Mills et al. 2020). The peripheral mechanisms involved in orofacial pain might be attributed to changes in macrophage phenotype in the trigeminal ganglion (TG), mediated by sympathetic outflow (Mai et al. 2022). Additionally, interactions between neurons and non-neuronal cells in the primary sensory ganglion appear to contribute to this process (Chamessian and Zhang 2016). Mechanical allodynia is triggered by the activation of touch receptors or sensitized mechanoreceptors (Woolf 2010), whereas cold allodynia results from changes in ion channel activity, including reduced potassium currents or heightened expression of receptors such as TRPM8 (Piña et al. 2024), both phenomena suggesting central sensitization.

The TMJ arthritis model employed in this investigation with CFA containing *Mycobacterium tuberculosis* causes arthritis and degradation of the TMJ cartilage and subchondral bone via macrophage immune response (Xiang et al. 2021). The resulting synovitis, bone resorption, degeneration, and articular pain are triggered by the synthesis and release of pro-inflammatory mediators. CFA alters the fibrocartilage of the TMJ articular surface and affects the biomechanical properties of the disc (Wang et al. 2012; Zhao et al. 2024). Togni et al. (2018) reported notable degradation of the articular cartilage and subchondral bone, replacement by granular tissue in the bones, a reduction in cartilage thickness, and the migration of multinucleated osteoclastic cells in the superior region of the affected condyle under the same pain paradigm. Each phase, including the inflammatory, contributes to TMJ hypernociception, as demonstrated by previous studies in rats using CFA in the TMJ (Kroeff et al. 2025; Marini et al. 2025; Scarabelot et al. 2018; Vicenzi et al. 2025).

The CFA-induced TMJ arthritis model in this study resulted in long-term orofacial mechanical allodynia, observed up to day 17, characterized by the perception of typically non-painful mechanical stimuli as painful. Previous studies in our laboratory have documented orofacial mechanical allodynia following intra-TMJ CFA injection for a duration of up to 22 days in both Sprague-Dawley and Wistar rats (Scarabelot et al. 2018; Vicenzi et al. 2025). The trigeminal nerve, a key component of the trigeminal system linked to chronic orofacial pain, has specific features and functions that include the provision of all facial special sensations, differing from those in the spinal nerves (Han et al. 2023). The structural and functional characteristics of orofacial pain disorders reduce their similarity to conditions found in the spinal regions. Animals exhibiting reduced baseline mechanical thresholds displayed an augmented mechanical nociceptive response at D17, emphasizing the significance of baseline features in influencing nociceptive levels.

Conversely, cold allodynia serves as an important measure for assessing pain behavior. Clinical neuropathic pain conditions are characterized by severe cold allodynia, as demonstrated in a study where all patients with post-traumatic neuralgia exhibited this symptom. In experimental contexts, cold allodynia is evaluated through the acetone evaporation test, which assesses grooming behavior as an indirect measure of this condition (Ruan et al. 2018). Thermal evaluations of both heat and cold are useful for neuropathic pain models; however, heat is recommended for inflammatory pain models, as it provides a more suitable assessment for this type of pain. Allchorne et al. (2005) demonstrated cold allodynia in Sprague-Dawley rats subjected to various neuropathic pain models, observed from 2 to 35 days following spared nerve injury surgery. Conversely, the authors indicated that rats exhibiting inflammation in the hind paw induced by CFA showed decreased paw withdrawal latency compared to naïve controls across all tested temperatures in the cold plate test (15, 10, and 5 °C) at 48 and 72 h following CFA administration. The current study corroborates that early cold allodynia was observed 72 h post-CFA injection (D3) and was no longer present by D17, indicating a transient manifestation of facial cold allodynia in CFA-induced TMJ arthritis in rats. An additional study utilizing streptozotocin (STZ)-induced diabetic rats to assess neuropathic pain revealed a higher frequency of nociceptive behaviors after acetone application, persisting for up to 4 weeks (Nam et al. 2014). In contrast, Jesus et al. (2022), utilizing the same STZ model, did not observe differences in the weekly assessed responses of diabetic animals in the cold plate test or acetone test over a four-week period. The data indicate that, in addition to the importance of the pain model in relation to cold allodynia, the animal lineage is a critical factor. Nam et al. employed Sprague-Dawley rats, while Jesus et al. (2022) utilized Wistar rats, comparable to the present study, despite variations in pain types (neuropathic versus inflammatory). In addition, a negative association was observed at D3 between mechanical nociceptive threshold (von Frey test) and thermal nociceptive threshold (grooming time in the Acetone test). It is noteworthy that CFA injection demonstrated efficacy, as animals in the Pain group significantly differed from the Control group, while the Sham group displayed a response that was intermediate between the Control and Pain groups, suggesting a residual effect of the saline injection. Increased grooming time is associated with a reduced mechanical nociceptive threshold, indicating a more intense nociceptive response. The grooming duration exhibited a positive correlation with IL-4 levels in the spinal cord. The findings demonstrate that the arthritis model employed in this study effectively replicated chronic inflammatory orofacial pain.

The increased levels of IL-4 and TNF-α observed in the spinal cord, as opposed to the brainstem, likely indicate local differences in neuroanatomical function and neuroimmune interactions in the orofacial pain model. The brainstem trigeminal nuclei primarily function as initial nociceptive relays, while the spinal cord and the trigeminal nucleus caudalis, which are functionally similar to the dorsal horn, represent significant sites of sustained glial activation and central sensitization (Bradesi, 2010; Schulte et al. 2015). The activation of C and Aδ fibers leads to sustained responses in microglia and astrocytes, which in turn results in elevated levels of pro-inflammatory cytokines, including TNF-α, as well as compensatory anti-inflammatory mediators like IL-4. At 17 days post-induction, sustained neuroimmune activity indicates mechanical allodynia and suggests a transition to a subacute-to-chronic pain state primarily influenced by central mechanisms. The lack of observable cytokine alterations in the brainstem likely indicates the localized nature of trigeminal neuroinflammation, which may be obscured when larger brainstem areas are examined as a whole tissue sample.

It is important to note that, while neuroinflammation may initially protect the CNS, prolonged or excessive activity can have detrimental effects (Xu et al. 2018). TNF-α is elevated in several inflammatory conditions, including trauma, sepsis, and rheumatoid arthritis (Gharamti et al. 2022; Hassany et al. 2024; Liu and Tang 2014), and contributes to central sensitization through both TNF-α receptor types (Zhang et al. 2010). Its pronociceptive role underlies hyperalgesia in arthritis and temporomandibular disorders (Cardoneanu et al. 2022; König et al. 2013). Increased TNF-α in the TG and Sp5C after intra-TMJ CFA injection further supports its involvement in TMJ inflammatory pain (Bai et al. 2018). Although brainstem TNF-α levels remained unchanged, their correlation with spinal levels highlights the cytokine relevance in arthritis pathophysiology, suggesting that TNF-α inhibition may yield antinociceptive effects (Öz and Şi̇Mşek 2022).

On the other hand, IL-4 is known for its dual functions; in addition to its anti-inflammatory properties, IL-4 can synergistically enhance pro-inflammatory cytokines such as TNF-α (Vanderwall and Milligan 2019). A previous study demonstrated increased IL-4 mRNA levels in the whole blood of patients with rheumatoid arthritis, comparable to levels found in synovial fluid and plasma (Bahlas et al. 2019; Schlaak et al. 1996). Kokkonen et al. (2010) observed increased IL-4 levels in rheumatoid arthritis patients prior to disease onset, a finding that was also observed in our rat model of TMJ arthritis, though in the central CNS. The findings suggest that peripheral IL-4 may stimulate a reciprocal neuroinflammatory response, highlighting a significant translational aspect of these results and supporting the findings of the current study regarding chronic inflammatory pain. The data also support the hypothesis that peripheral IL-4 levels might act as a specific biomarker for cytokine activity in the CNS, highlighting the complex relationships between central pro- and anti-inflammatory cytokines. There were positive correlations between the levels of TNF-α in both the brainstem and spinal cord and spinal cord IL-4 levels. Furthermore, although central IL-10 levels did not show significant differences between groups, spinal cord IL-10 concentrations exhibited a significant correlation with IL-4 levels in both the brainstem and spinal cord, underscoring a coordinated regulatory network that may be crucial for the precise modulation of neuroimmune responses.

This study points out the antinociceptive effects of RES during the latter stages of inflammation. The analgesic efficacy of RES is dose-dependent, showing less effectiveness at 20 mg/kg/day compared to higher doses. Furthermore, all administered RES doses significantly reduced the elevated TNF-α levels in the spinal cord of arthritic rats, accordingly confirming its anti-inflammatory effect. As discussed above, an elevation in central IL-4 levels was noted in arthritic rats, which was significantly boosted by 80 mg/kg/day RES, suggesting that higher doses are necessary to stimulate the anti-inflammatory signaling. In accordance, a preclinical study demonstrated that a high dosage of RES (200 mg/kg) administered over 14 days enhanced IL-4 receptor-mediated anti-inflammatory responses in the spinal cord of rats, potentially aiding in the reduction of central sensitization after peripheral nerve injury (Xu et al. 2018), highlighting its role in regulating the inflammatory response and providing neuroprotection (Zhang and An 2007). It is important to note that the effects of RES are significantly influenced by variables including dose, route of administration, duration of exposure, and pain model employed (Bejenaru et al. 2024; Szymkowiak et al. 2024; Wu et al. 2025).

The human equivalent dose of RES utilized in this study is 12.97 mg/kg (Reagan et al. 2007). Rodent trials have utilized RES at doses between 4 and 300 mg/kg (Khorshidi et al. 2020). Conversely, Singh and Vinayak (2016) demonstrated significant antinociceptive effects of RES in the same pain model at oral doses of 50 and 100 mg/kg. RES functions by inhibiting classical pro-inflammatory pathways, such as NF-κB, and promoting a Th2-type immune response associated with the resolution of inflammation (Moussa et al. 2017). A prior study demonstrated that seven-day consecutive intrathecal administration of RES alleviated mechanical allodynia in rats throughout the observation period. Simultaneously, treatment with RES reduced the synthesis of pro-inflammatory mediators TNF-α, IL-1β, and IL-6 and inhibited the expressions of phospho-JAK2 and p-STAT3 in the lumbar spinal dorsal horns on postoperative day 7 (Han et al. 2023). This last study, alongside others utilizing neuropathic and inflammatory experimental pain models, supports the neuromodulatory effects of RES also identified in the current investigation, demonstrating its anti-inflammatory and neuromodulatory properties, which may potentially explain the observed analgesic effects. Moreover, regarding potential adverse effects, clinical studies demonstrated that high doses of resveratrol, specifically 500, 1,000, 2,500 or 5,000 mg/day over 29 days led to mild to moderate gastrointestinal symptoms only at elevated doses, with no significant serum toxicity detected (Brown et al. 2010). A 90-day randomized clinical trial involving older adults, which administered 300 mg/day and 1,000 mg/day, found no significant adverse effects or changes in serum levels (Anton et al. 2014). The data indicate that RES has an extensive therapeutic window and lacks significant adverse effects.

Chronic pain produced by the TMJ arthritis model did not affect behavioral parameters evaluated in the OF and EPM tests, supporting findings from our previous study using the same pain model, yet assessed on day 19 post-CFA (Marini et al. 2025). However, another study conducted recently in our lab revealed that orofacial arthritic rats exhibited a lower anxiety-like behavioral index at D26, in the absence of mechanical allodynia (Vicenzi et al. 2025). One could conclude that the timing of evaluations following the induction of the pain model is essential for behavioral outcomes. Moreover, the dosages of RES administered were likely insufficient to elicit significant behavioral effects, especially in light of the pharmacokinetic properties associated with the compound’s bioavailability. The oral absorption of resveratrol was estimated to be at least 75%; however, its low oral bioavailability, rapid hepatic metabolism, and limited CNS penetration restrict the achievement of sufficient therapeutic concentrations in target tissues (Smoliga and Blanchard 2014; Walle et al. 2004). Managing orofacial pain poses clinical and experimental challenges owing to the distinct anatomical and functional characteristics of the trigeminal nerve, which may affect the effectiveness of analgesic and anti-inflammatory treatments. Future research will encompass higher doses of RES and pharmacological approaches to improve bioavailability, including nanostructured systems and alternative experimental paradigms.

The current study has some limitations: first, only male subjects were used; this intentional selection aimed to reduce the confounding effects of hormonal fluctuations on the nociceptive threshold when using females. Female rats may have different behavioral and biomarker responses; we recently conducted a similar study in ovariectomized female rats using the TMJ arthritis model (Kroeff et al. 2024). Second, the lack of statistically significant differences in certain comparisons might result from insufficient statistical robustness, attributed to the substantial data dispersion commonly observed in animal experimentation. Third, tramadol administered during the early inflammatory phase of CFA may be a significant confounding factor influencing both early pain behavior and cytokine responses. On the other hand, this study employed an animal model of chronic pain that developed progressively, with no surgical intervention; however, it is very acutely painful, and post-induction analgesia with tramadol was used to prevent this post-procedure pain, an acute condition, the same methodology used in previous studies by our research group (Kroeff et al. 2025; Marini et al. 2025).

Ultimately, these findings, particularly the advantageous effects on mechanical allodynia and the levels of central TNF-α and IL-4, offer robust experimental evidence for the immunomodulatory capacity of RES in inflammatory pain scenarios, despite the behavioral outcomes being modest and statistically insignificant. These results indicate that the RES has therapeutic promise as an adjuvant in the management of painful inflammatory diseases, including TMJ disorders.

## Conclusion

This study demonstrates that the CFA-induced TMJ arthritis model in rats accurately replicates chronic orofacial pain, marked by persistent mechanical allodynia, temporary cold allodynia, and central neuroimmune modifications. The repeated administration of resveratrol elicited a dose-dependent antinociceptive response, with elevated doses completely improving nociceptive thresholds. The partial effect noted at RES20 indicates that inadequate dosing might not be sufficient to mitigate chronic pain, underscoring the necessity for dose optimization. RES also influenced neuroimmune responses: only RES80 significantly increased IL-4 levels, while TNF-α increases were mitigated at all dosages, corroborating the compound’s anti-inflammatory characteristics. These findings emphasize the role of both peripheral and central pathways in the onset and persistence of TMJ inflammatory pain and underscore the significance of immune regulation in nociception. The significant analgesic and anti-inflammatory properties of RES in this chronic inflammatory orofacial pain paradigm support its potential translational use as an adjuvant in managing painful inflammatory diseases, including temporomandibular joint (TMJ) disorders.

## Data Availability

The data that support the findings of this study are not openly available due to reasons of sensitivity and are available from the corresponding author upon reasonable request.

## **Abbreviations**

TMD: Temporomandibular dysfunction
TMJ: Temporomandibular joint
NSAIDs: Nonsteroidal anti-inflammatory drugs
RES: Resveratrol
TNF-α: Tumor Necrosis Factor alpha
IL-1β: Interleukin 1 beta
CREAL: Laboratory animal reproduction and experimentation center
UFRGS: Universidade Federal do Rio Grande do Sul
HCPA: Hospital de Clínicas de Porto Alegre
CFA: Complete Freund’s Adjuvant
PO: Per oral
VF: von Frey
OF: Open field
EPM: Elevated plus maze
EOA: Entries into the open arms
ECA: Entries into the closed arms
TE: Total number of entries
TOA: Time spent in open arms
TCA: Time spent in closed arms
PHD: Protected head dips
UDH: Unprotected head dips
PBS: Phosphate buffered saline
IL-4: Interleukin 4
IL-10: Interleukin 10
SPSS: Statistical package for the social sciences
SD: Standard deviation
IQR: Interquartile range
CT: Full / Total control
BS: Brainstem
SC: Spinal Cord
CNS: Central nervous system
TG: Trigeminal Ganglion
STZ: Streptozotocin
